# Characterization of Taurocholic Acid Binding With Insulin for Potential Oral Formulation Using Different Methods

**DOI:** 10.1002/elps.8139

**Published:** 2025-04-16

**Authors:** Chang Sun, Shuanghao Wang, Huihui Li, David Da Yong Chen

**Affiliations:** ^1^ State Key Laboratory of Analytical Chemistry for Life Science National and Local Joint Engineering Research Center of Biomedical Functional Materials Jiangsu Collaborative Innovation Center of Biomedical Functional Materials Changzhou Institute of Innovation and Development School of Chemistry and Materials Science Nanjing Normal University Nanjing People's Republic of China; ^2^ Jiangyan High School of Jiangsu Province Taizhou People's Republic of China; ^3^ Jiangsu Key Laboratory for Biosensors Institute of Advanced Materials (IAM) Nanjing University of Posts & Telecommunications Nanjing People's Republic of China; ^4^ Department of Chemistry University of British Columbia Vancouver British Columbia Canada

**Keywords:** capillary electrophoresis, insulin, interaction, mass spectrometry, taurocholic acid

## Abstract

In diabetes management, oral formulation of insulin (INS) has the potential to improve safety, convenience, and patient‐centered care compared to subcutaneous injections. However, its bioavailability remains limited, necessitating improved delivery strategies. Recent clinical trials indicate that taurocholic acid (TCA) can enhance the bioavailability of oral INS as an absorption enhancer. In this work, electrospray ionization mass spectrometry (ESI‐MS) analysis revealed the formation of 1:1–1:4 INS–TCA complexes. MS/MS was used to explore the fragmentation pathway of complex ions and confirm binding stability in the gas phase. Circular dichroism spectra showed no clear conformational change in INS upon TCA binding, even though TCA enhanced INS's structural stability. Using Taylor dispersion analysis (TDA), we determined the diffusion coefficient and hydrodynamic radius of INS and its complexes. TCA binding was observed to increase INS size in both the 1:1 and 1:2 INS–TCA complexes. The binding constant of INS and TCA (1.3 × 10^3^ L/mol) with approximately five binding sites was obtained via pressure‐assisted capillary electrophoresis frontal analysis. Molecular docking simulations indicated that TCA binds to external binding sites on the INS B chain (near Ser‐B9, Glu‐B13, and Phe‐B24 residues), consistent with ESI‐MS and TDA results. These findings suggest that TCA binding may enhance INS absorption and increase the bioavailability of oral INS therapy.

**Abbreviations**
CD: circular dichroism
INS: insulin
MOE: Molecular Operating Environment
PACE‐FA: pressure‐assisted capillary electrophoresis frontal analysis
PDB: Protein Data Bank
TCA: taurocholic acid
TDA: Taylor dispersion analysis

## 1 Introduction

Diabetes, a chronic metabolic disorder, is one of the most challenging health issues of the 21st century. The metabolism of sugar, fat, and protein is interrupted, affecting the water and electrolyte balance [1, 2]. Although diabetes currently has no cure, it can be effectively managed through lifestyle changes, insulin (INS) therapy, and oral antidiabetic drugs. Existing diabetes treatments have gradually evolved to focus on blood sugar reduction, blood pressure control, lipid regulation, and anticoagulation. Primary methods for lowering blood sugar levels include oral hypoglycemic drugs [3] and INS injections [4, 5]. However, complications associated with oral hypoglycemic drugs can challenge patients’ daily activities, negatively impacting their quality of life [6, 7]. INS, a protein peptide drug, can be degraded by proteolytic enzymes in the gastrointestinal tract [8]. If taken orally without specialized delivery systems, its bioavailability is only 0.1%–2%. This is due to enzymatic and chemical barriers in the stomach, degradation by proteolytic enzymes in the intestine, and poor permeability across the intestinal epithelium [9]. Although subcutaneous injections are common in clinical practice, multiple daily injections cause inconvenience and pain to patients [10, 11, 12]. Consequently, there is an urgent need for safe, convenient, and effective oral INS options for diabetes management.

Oral INS therapy remains a global challenge due to three major obstacles: (1) INS's relatively high molecular weight, which limits its ability to pass through the intestinal mucosa [4]; (2) degradation by proteases in the digestive tract [13, 14]; and (3) the influence of body pH and temperature on INS stability and function [4]. An ideal oral INS delivery system should release its contents in the optimal target area and pH, maintain it at the ideal site long enough for complete release, enable absorption by the intestinal epithelium, and produce consistent therapeutic effects [15]. Presently, many pharmaceutical strategies focus on designing novel carrier‐mediated oral INS delivery systems [16, 17, 18], as well as enhancing INS absorption and inhibiting protease degradation [19, 20]. Al‐Remawi et al. prepared a lipid amino acid nanocarrier based on the interaction between L‐arginine and oleic acid with a Tween 80 adjuvant. This formulation achieved a 4% reduction in blood glucose 80 h after oral INS administration [21]. They also created an INS‐chitosan complex with phospholipid liposomes as a novel carrier for oral INS delivery, which demonstrated longer lasting hypoglycemic effects compared to subcutaneous INS injections [22]. Li et al. advanced oral INS administration by using carboxymethyl chitosan nanoparticles, achieving high bioavailability and sustained release, with a relative pharmacological bioavailability of 14.71% [23]. Momoh et al. developed an innovative INS‐loaded micro‐location system utilizing snail mucin, which encapsulated INS within a water‐in‐oil core stabilized with Tween 80. This formulation effectively reduced blood glucose levels for 8 h post‐oral administration [24]. Mooranian et al. prepared various insoluble alginate esters using polyacrylate polymers encapsulated in the antioxidant drugs: probucol and taurocholic acid (TCA). The results showed that TCA, a bile acid, facilitated the oral administration of antidiabetic drugs [25].

**FIGURE 1:**
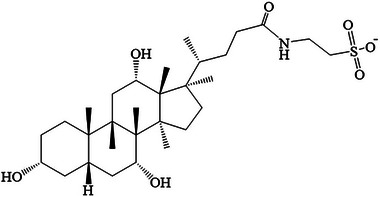
The chemical structure of TCA.

Absorption enhancers promote drug absorption by increasing the cross‐cellular transport of INS. Their mechanisms may include reducing mucus viscosity, altering membrane fluidity, increasing membrane permeability, and expanding the number of tightly‐connected pores [26]. Common absorption enhancers include Tween, azone, citric acid, salicylic acid, and ethylenediamine tetraacetic acid [27, 28]. Bile acids, abundantly present in the intestine, liver, skeletal muscles, and pancreas, are known to inhibit nutrient‐stimulated cholecystokinin release, gallbladder contraction, and small intestinal transit. Recent research has highlighted that bile acids are essential in blood glucose regulation. The use of bile acids as INS absorption enhancers has proven more effective, enhancing oral absorption and the hypoglycemic effects of INS [29]. Absorption enhancers and enzyme inhibitors are often added as adjuvants to carriers such as microcapsules, liposomes, and nanoparticles, thereby improving the oral bioavailability of INS. Recent clinical trials have demonstrated that TCA (Figure 1), as an absorption enhancer in oral formulations, can significantly improve the bioavailability of oral INS [30, 31]. While the transport mechanisms remain largely unknown, this has prompted us to explore the interaction between TCA and INS. Consequently, there is a need for a fast and effective analytical strategy that enables comprehensive analysis of the interaction mechanism between TCA and INS.

In this study, the binding capacity and stoichiometry of TCA with INS were rapidly evaluated using electrospray ionization mass spectrometry (ESI‐MS). The stability of the INS‐TCA non‐covalent complex ions was preliminarily investigated by tandem mass spectrometry (MS/MS) through collision‐induced dissociation (CID). Circular dichroism (CD) spectroscopy helped analyze the structural changes in INS upon TCA addition. The diffusion coefficient and radius of INS before and after TCA binding were determined using Taylor dispersion analysis (TDA). To accurately determine their binding strengths, a pressure‐assisted capillary electrophoresis frontal analysis (PACE‐FA) method was developed, calculating the stoichiometry and binding constants of the native‐state binding pair in free solution via nonlinear regression analysis. Finally, a binding model was constructed using molecular docking simulations, showing that TCA binds to INS and stabilizes its spatial structure. This study suggests that TCA acts as an absorption enhancer that promotes drug absorption through non‐covalent interactions with INS.

## 2 Materials and Methods

### 2.1 Materials

Human INS (≥98%) was purchased from Tonghua Dongbao Pharmaceutical Co., Ltd. (Jilin, China). Chromatography‐grade ethanol and methanol were obtained from Sigma‐Aldrich (Shanghai, China). Chromatography‐grade ammonium acetate, TCA, cholesterol, lecithin, and glycinocholic acid were procured from Aladdin Biochemical Technology Co. Ltd. (Shanghai, China). Analytical‐grade sodium dihydrogen phosphate, phosphoric acid, hydrochloric acid (HCl), sodium hydroxide (NaOH), and hydroxypropyl cellulose (HPC) were sourced from Wanqing Glass Instrument Co., Ltd. (Nanjing, China).

### 2.2 ESI‐MS

An Orbitrap Fusion Lumos Tribrid mass spectrometer (Thermo Fisher Scientific) was used for the ESI‐MS analysis. A 10 µM INS sample was diluted in 2 mM HCl and mixed with TCA (1:3.5 to 1:20 in molar ratio). This mixture was incubated for 10 min at 37°C to attain chemical equilibrium. The sample was injected into the mass spectrometer at a flow rate of 3.0 µL/min. The capillary voltage was set to 2.5 kV when the ion transfer tube temperature reached 300°C. The mass spectra were fully scanned at a resolution of 60,000 in the *m/z* range of 150–4000. CID was used to assess INS–TCA complex stability and its fragmentation pathway in MS/MS mode. Data were analyzed using Xcalibur 4.0 (Thermo Fisher Scientific, USA).

### 2.3 CD Spectroscopy

CD experiments were performed using a Chirascan CD spectrometer (Applied Photophysics Ltd., England). The samples for CD measurement consisted of 60 µM INS at 2 mM HCl (pH 3.5) and its mixture solution upon TCA addition (INS:TCA = 1:5 in molar ratio). The CD spectra were scanned from 190 to 260 nm, with each sample measured three times (0.1 cm path length). A CD melting experiment was conducted in the temperature range of 25–92°C, with a melting rate of 2.0°C/min. Data processing was performed using Chirascan software (Applied Photophysics, UK).

### 2.4 TDA

TDA experiments were performed using a PA 800 Plus capillary electrophoresis instrument (Scientific, Framingham, MA, USA) equipped with a photodiode array (PDA) detector. The fused silica capillary tube (50 cm × 50 µm id, 360 µm od, the effective length of 40 cm) purchased from Polymicro Technologies (Phoenix, USA) was rinsed sequentially with 0.1 M NaOH for 8 min, 0.1 M HCl for 8 min, H_2_O for 8 min, air for 5 min, and then equilibrated with the running buffer (2 mM HCl) overnight. The sample was injected at 2 psi (13.8 kPa) for 5 s into a capillary prefilled with running buffer and then pushed through at a mobilization pressure of 2 psi (13.8 kPa). Each sample was incubated for 30 min at 37°C and then analyzed four times. The detection wavelength was set at 210 nm, and the capillary temperature was maintained at 25°C. Origin 8.5 was employed for software data processing (more details in the Supporting Information).

### 2.5 PACE‐FA

PACE‐FA experiments were performed using a PA 800 Plus capillary electrophoresis instrument (Scientific, Framingham, MA, USA) equipped with a PDA detector. The capillary (50 cm × 50 µm id, 360 µm od) with an effective length of 40 cm was coated with HPC to minimize the adsorption of analyte molecules on the inner wall. The capillary was rinsed at 40 psi (275.8 kPa) with methanol (30 min), deionized water (30 min), 0.1 M sodium hydroxide solution (30 min), deionized water (30 min), 5% (w/w) HPC water solution (30 min) sequentially, and an air‐drying step (60 min). After the rinsing and drying steps, the coated capillary was baked in a chromatographic oven under with a temperature program (heating from 60°C to 140°C at a rate of 5°C/min, then 140°C for 60 min) with N_2_ at 60 psi (413.7 kPa). Prior to use, the capillary was washed with the background electrolyte (15 mM NaH_2_PO_4_‐H_3_PO_4_, pH of 3.5) at 10 psi (69.0 kPa) for 30 min. Samples were injected at 1 psi (6.90 kPa) for 90 s at the cathodic part, and the analytes were separated at a voltage of +15 kV with a hydrodynamic pressure of +0.7 psi (+4.83 kPa). The detection wavelength was 210 nm and the temperature was 25°C. Each sample was tested in triplicate. Origin 8.5 was used for data processing.

### 2.6 Computer Simulation

For molecular docking simulation, the Protein Data Bank (PDB) (www.rcsb.org) provided the three‐dimensional (3D) structure of INS (PDB ID: 1MSO). The simulations were performed using the Molecular Operating Environment 2015.10 (MOE, Chemical Computing Group Inc., Canada). Before docking, crystal water molecules were removed and hydrogen atoms were added. One INS monomer was retained in the INS hexamer structure, while the other five monomers were removed. The 3D structure of TCA was mapped using ChemDraw 18.0 (CambridgeSoft, USA), followed by hydrogenation and energy minimization. Docking calculations for the INS‐TCA complex were carried out using the DOCK module of MOE. During the docking process, the Amber 10 EHT force field was applied to determine the optimal position for binding. Finally, the London dG scoring function was used for conformational scoring. The docking results were ranked based on binding energies and their corresponding scores. To visualize the results, the most reasonable docking conformation was selected and exported in PDB format, which could be viewed using the PyMOL software (DeLano Scientific LLC, USA).

## 3 Results and Discussion

### 3.1 Rapid Evaluation of the Binding of TCA to INS by ESI‐MS

**FIGURE 2:**
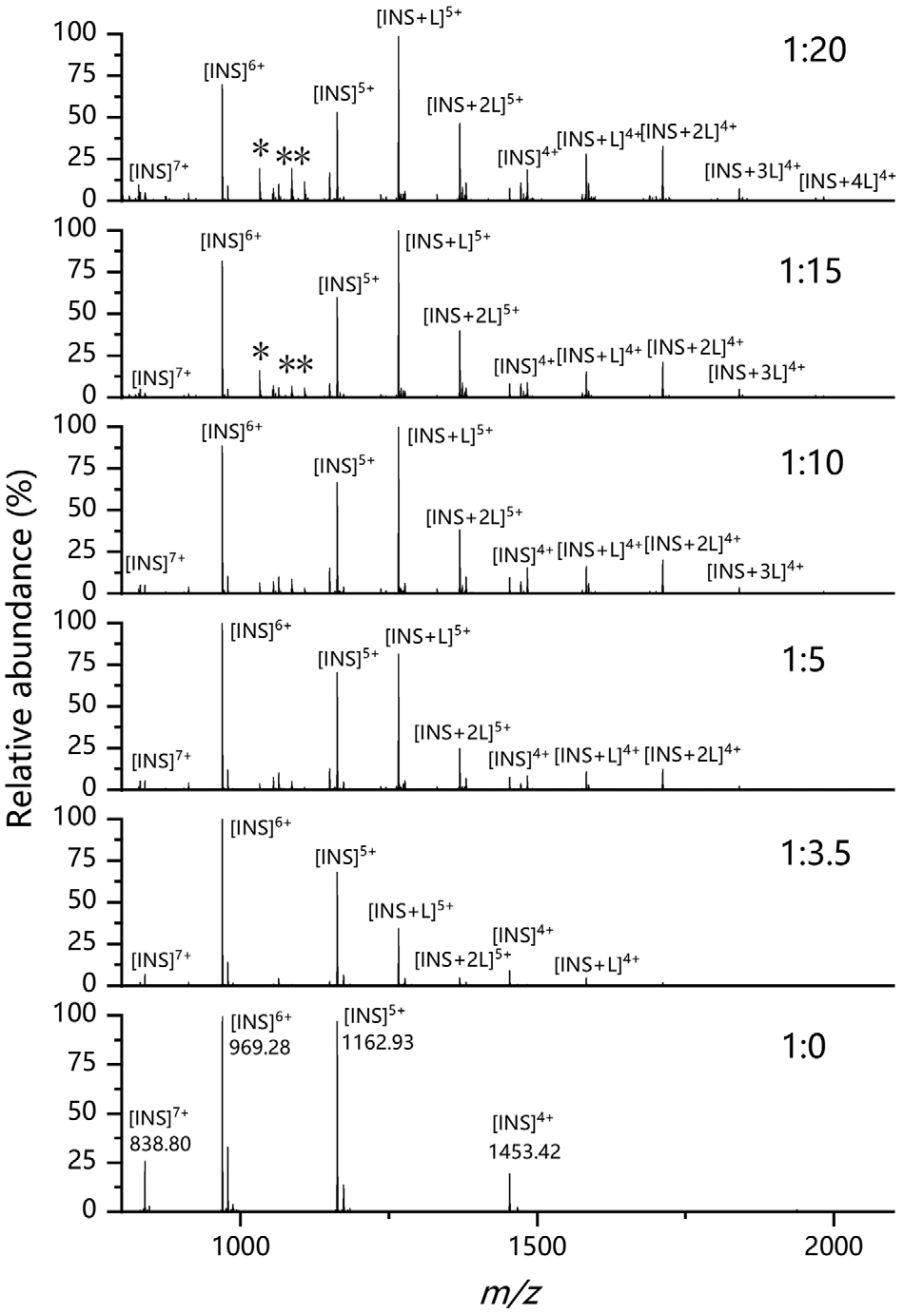
ESI‐MS spectra of 10 µM insulin mixed with TCA at different molar ratios of 1:0 to 1:20 with *m/z* ranging from 800 to 2100. * and ** indicate the TCA dimer and its adduct ions binding with H_2_Os ([2L]^+^ and [2L+3H_2_O]^+^ at *m/z* 1032.12 and 1086.06, respectively).

ESI is a soft ionization technique for investigating non‐covalent interactions between biological macromolecules and ligands with MS, and has been widely used to analyze protein–ligand interactions [32, 33]. A rapid ESI‐MS binding assay for INS‐TCA was used to assess the binding ability of TCA as an INS absorption enhancer. Under optimized conditions, INS and TCA mixtures with different molar ratios (1:0–1:20) were analyzed using direct injection mass spectrometry. Figure 2 displays the mass spectra obtained at *m/z* values ranging from 800 to 2100. The MS spectra revealed INS ions with charges ranging from 4+ to 7+ ([INS]*^n^* ^+^, *n* = 4 to 7, *m/z* = 1453.42, 1162.93, 969.28, and 838.80, respectively). With the addition of TCA, peaks corresponding to the INS‐TCA complex ions appeared in the spectra. When the INS:TCA molar ratio reached 1:3.5, the relative intensity of the 1:1 binding complex ion [INS+L]^5+^ (*m/z* = 1266.25) was approximately 35%. As the concentration of TCA increased, the relative intensity of the 1:1 complex ions gradually increased, and 1:*n* complex ions (*n* = 2–4) were detected. In the MS spectrum of the INS/TCA mixture at a 1:20 molar ratio, [INS+L]^5+^ was the base peak, with 1:2 to 1:4 binding complex ions also appearing, such as [INS+2L]^5+^ (*m/z* 1368.66) and [INS+mL]^4+^ (*m* = 2 to 4, *m/z* = 1710.57, 1839.64, and 1968.48, respectively). However, the relative intensities of these 1:2 to 1:4 complex ions did not exceed 50%, which was lower than that of the 1:1 complex ion. These results suggest that TCA primarily interacts with INS in a 1:1 stoichiometry, with weaker interactions observed in 1:2 to 1:4 stoichiometries. When the INS:TCA molar ratio reached 1:15 and 1:20, the dimer ion of TCA and its adduct binding with H_2_O ([2L]^+^ and [2L+3H_2_O]^+^ at *m/z* 1032.12 and 1086.06, respectively) were present in the spectra, probably due to the nature of ESI at high concentrations of analytes [34].

### 3.2 Gas‐Phase Stability of the 1:1 INS‐TCA Complex by ESI‐MS/MS

**FIGURE 3:**
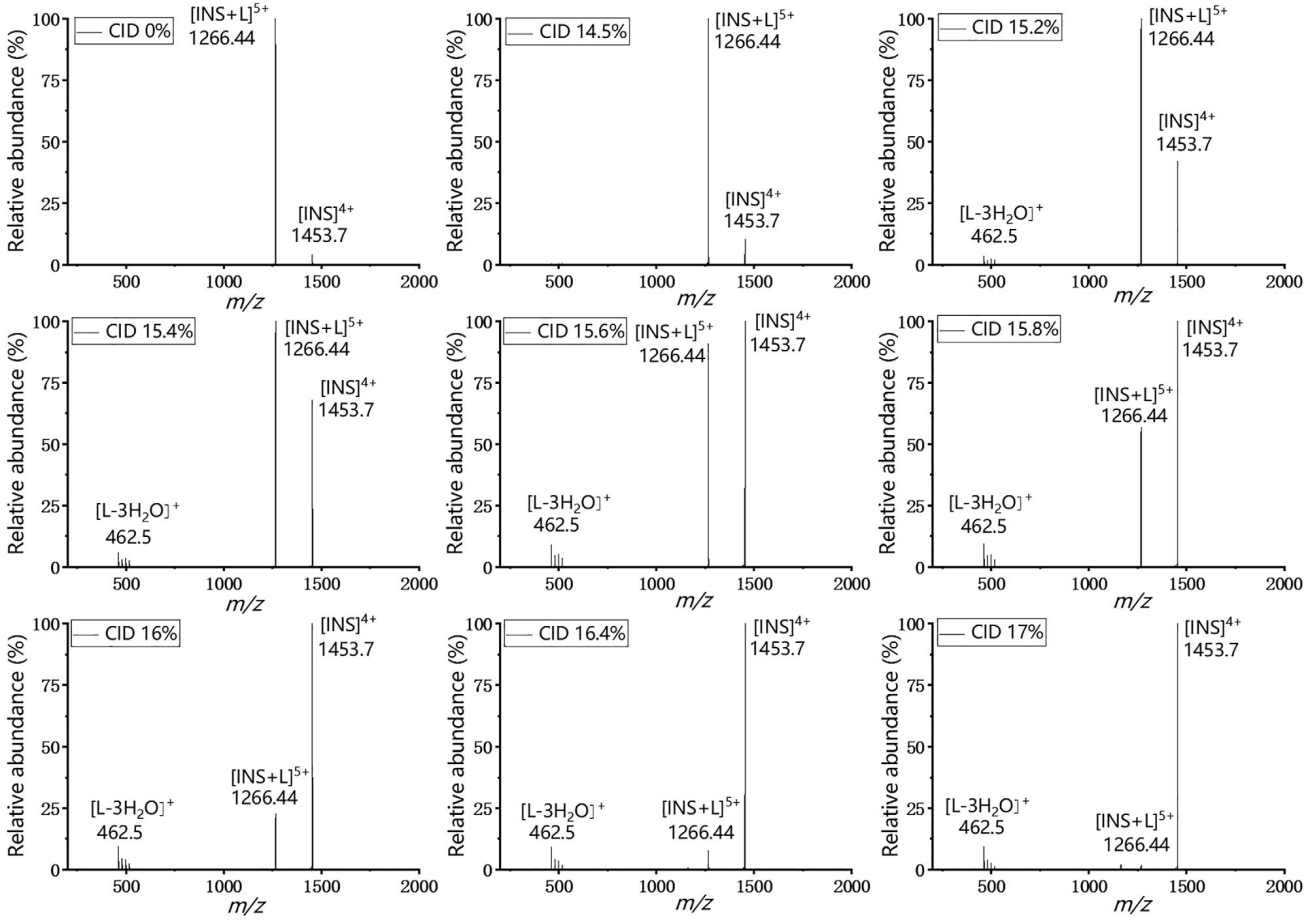
The MS/MS spectra of the 1:1 INS‐TCA complex ion [INS+L]^5+^ at *m/z* 1266.44 with CID energy of 0%–17%.

ESI‐MS/MS with CID was used to investigate the fragmentation pathway of the INS‐TCA complex ion and its stability in the gas phase. The ESI‐MS results suggested that TCA tends to bind to INS in 1:1 stoichiometry. The ESI‐MS/MS spectra of the complex ions provide further insights into how the TCA molecule binds to INS and whether the interaction is stable. As shown in Figure 3, energy‐dependent MS/MS data for the precursor 1:1 complex ion [INS+L]^5+^ (*m/z* = 1266.44) were acquired at increasing collision energies (between 0% and 17%).

As the collision energy increased to 14.5%, the complex ion began to dissociate and lose its guest TCA, generating [INS]^4+^ (*m/z* = 1453.70). At a collision energy of 15.2%, the intensity of the fragment ion [INS]^4+^ increased, with a series of [L]^+^ (*m/z* = 516.56) ion fragments appearing in the MS/MS spectrum. These additional ions showcased a loss of 1–3 H_2_O units (*m/z* = 498.54, 480.52, and 462.50). At 15.8% collision energy, the [INS]^4+^ ion became the base peak. At a higher collision energy of 17%, nearly all complex ions had fragmented, producing [INS]^4+^ as the base peak and weak TCA fragments (approximately 9% in intensity, *m/z* = 498.54, 480.52, and 462.50). ESI‐MS/MS results confirmed the presence of INS‐TCA complex ions in the gas phase, demonstrating their stability. The predominant fragmentation pathway of the 1:1 INS‐TCA complex ion through CID was dissociation into [INS]^4+^ and TCA ion fragments, with a loss of 1–3 H_2_O units. Studies using nuclear magnetic resonance and fluorescence quenching indicate that there is an external binding site with specific interactions between bound glucose and certain side chains on the INS surface [35]. In addition, docking and molecular dynamics simulations revealed that the hydrophobic pocket located between Val‐B2 and Leu‐B17 has a higher affinity for glucose compared to other regions of the INS surface [36]. Based on these findings, we hypothesized that TCA binds to the external binding sites of INS molecules.

### 3.3 Effect of TCA Binding on the Secondary Structure and Stability of INS Analyzed by CD Spectroscopy

**FIGURE 4:**
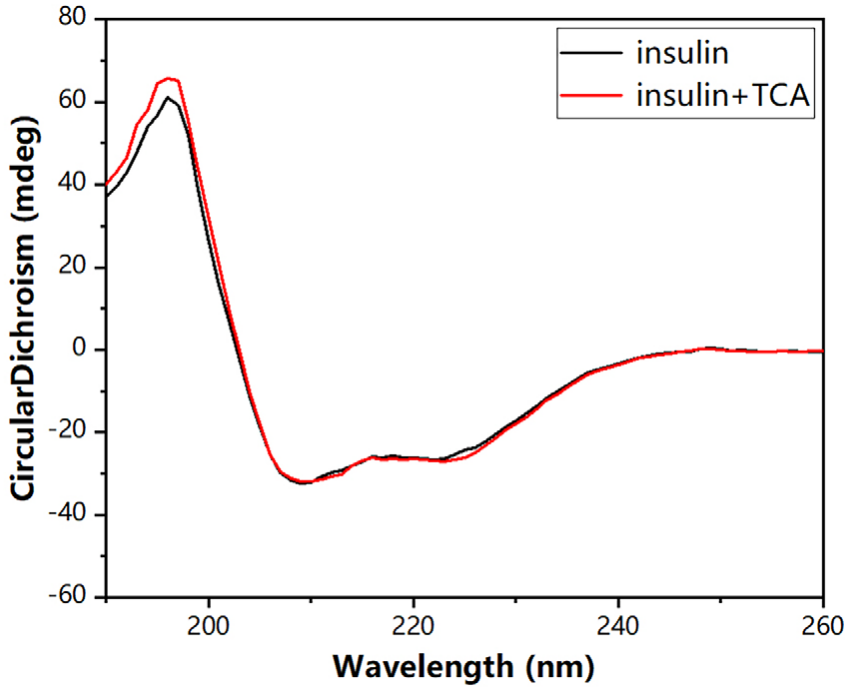
CD spectra of 60 µM insulin in the absence and presence of 500 µM TCA (2 mM HCl, pH 3.5) at 25°C.

The CD spectrum of 60 µM INS mixed with 300 µM TCA displayed a maximum at 196 nm and minima at 209 and 224 nm, showing minimal deviation from the spectrum of free INS (Figure 4). The estimated secondary structural content of INS comprised around 35% α‐helix, 16% β‐sheet, and 49% irregular structures. These findings were in close agreement with those of previous reports [37]. The analysis indicated that molar ellipticity ratios at 209 and 224 nm were approximately 1.27 before and after TCA binding, suggesting no noticeable quaternary conformational change in INS.

**FIGURE 5:**
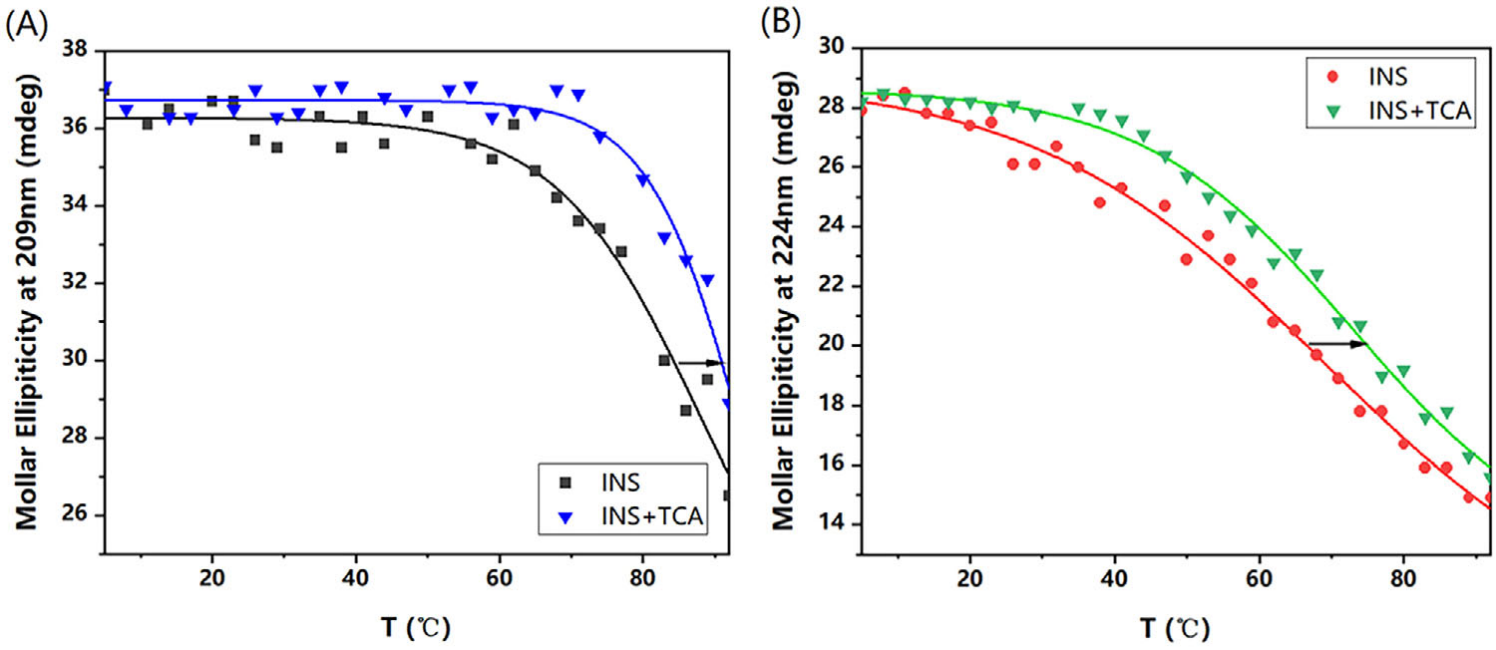
CD melting curves of insulin and insulin mixing with TCA in a molar ratio of 1:5 (2 mM HCl, pH 3.5) at 209 nm (A) and 224 nm (B).

To further explore the effect of TCA binding on the structural stability of INS in the solution phase, the apparent stabilization of INS by TCA was observed via melting experiments. Variations in the CD peaks at 224 and 209 nm were used to monitor alterations in the secondary structure of INS at different temperatures (Figure 5). The molar ellipticity of free INS gradually decreased as the temperature rose from 25°C to 92°C, with the melting temperature (*T*_m_) of INS at 209 nm being 88°C. For the INS–TCA complexes, a transition occurred at 95°C. Similarly, the TCA binding significantly increased the *T*_m_ value of INS from 69°C to 73°C observed at 224 nm. The melting point changed because the binding of TCA stabilized the spatial structure of INS.

### 3.4 Determination of Diffusion Coefficients and Hydrodynamic Radii of INS and Its Complexes Using the TDA Method

TDA is a recently developed technique for sizing analytes ranging from small and large molecules to micron‐scale particles and complexes. Herein, we utilized the TDA method to investigate the interaction between INS and TCA in nanoliter samples [38, 39]. The procedure, which only requires applying pressure to a thin tube or capillary, can be performed using a capillary electrophoresis (CE) instrument with a PDA detector. This approach enables the determination of diffusion coefficients and analyte sizes, providing valuable insights into the potential of TCA as an INS binder.

According to Taylor's theory, a plug containing the analyte in a capillary migrates under a Poiseuille laminar flow, leading to dispersion. Under certain conditions driven by steady pressure, the diffusion coefficient (*D*) of the analyte is related to temporal variance (*σ*_t_^2^) and the average migration time (*t*_d_), as shown in Equation (1) [40, 41]:

(1) $$D=\frac{R_{c}^{2}t_{d}}{24\sigma_{t}^{2}}\;$$

where *R*_c_ denotes the capillary radius. The Stokes–Einstein equation was used to determine the hydrodynamic radius *R*_h_ of the analyte, as shown in Equation (2) [42]:

(2) $$R_{h}=\frac{k_{B}T}{6\pi \eta D}\;$$

where *k*_B_ denotes the Boltzmann constant, *η* is the viscosity of the solvent in the capillary, and *T* indicates the temperature. According to Poiseuille's law, the linear velocity of the analyte in a laminar flow is inversely proportional to the viscosity of the solution [43]. When both the capillary size and the mobilizing pressure are held constant, the migration time of the analyte is directly proportional to the viscosity of the carrier liquid, as described by the following Equation (3):

(3) $$\;\;\frac{\eta }{\eta_{0}}=\left(\frac{PR_{c}^{2}t_{d}}{8L_{c}L_{d}}\right)/\left(\frac{PR_{c}^{2}t_{0}}{8L_{c}L_{d}}\right)=\frac{t_{d}}{t_{0}}$$

where *η*_0_ represents the viscosity of pure water and *t*_0_ denotes the migration time of the analyte in pure water, which serves as a reference. *L*_d_ and *L*_c_ are the capillary length from the injection end to the detector and the total length of the capillary, respectively. *P* is the mobilizing pressure. The viscosity of the solvent within the capillary can be measured using a CE instrument.

TDA measurements must satisfy two conditions to yield accurate results for the diffusion coefficient and hydrodynamic size of the analytes [44]. First, the migration time should be much longer than the characteristic time for analyte diffusion across the capillary radius. This condition is expressed as:

(4) $$t_{d}\gg \frac{3R_{c}^{2}}{80D\varepsilon }$$

In Equation (4), 𝜀 is the relative error that can be tolerated in determining *D*. The second condition is that longitudinal diffusion must be negligible compared to radial dispersion. To meet this condition, the Peclet number (*Pe*), which represents the ratio of contributions to mass transport by convection to those by diffusion, must exceed a certain value based on the desired error percentage of the diffusion coefficient. This condition is expressed in Equation (5).

(5) $$Pe=\frac{uR_{c}}{D}\;\gg \sqrt{\frac{48}{\varepsilon }}$$

where *u* denotes the linear velocity of the analyte. In all cases, the validity of the TDA conditions was verified, and the optimum conditions for performing TDA with INS were discussed. The operating parameters, including mobilizing pressure (1–5 psi) and capillary length (50, 60, and 70 cm) for INS, were investigated (Tables S1 and S2). For all TDA experiments presented in this study, the values of *t*_R_ >> 1.25 and *Pe* >> 40 were achieved; thus satisfying Taylor's conditions. The *R* ^2^ value (close to 1) is typically calculated by fitting the elution peak using a Gaussian function. To obtain a small standard deviation of the diffusion coefficient and a high value of *R* ^2^, the subsequent TDA experiments employed a mobilizing pressure of 2.0 psi (13.8 kPa) in a 50‐cm capillary.

**FIGURE 6:**
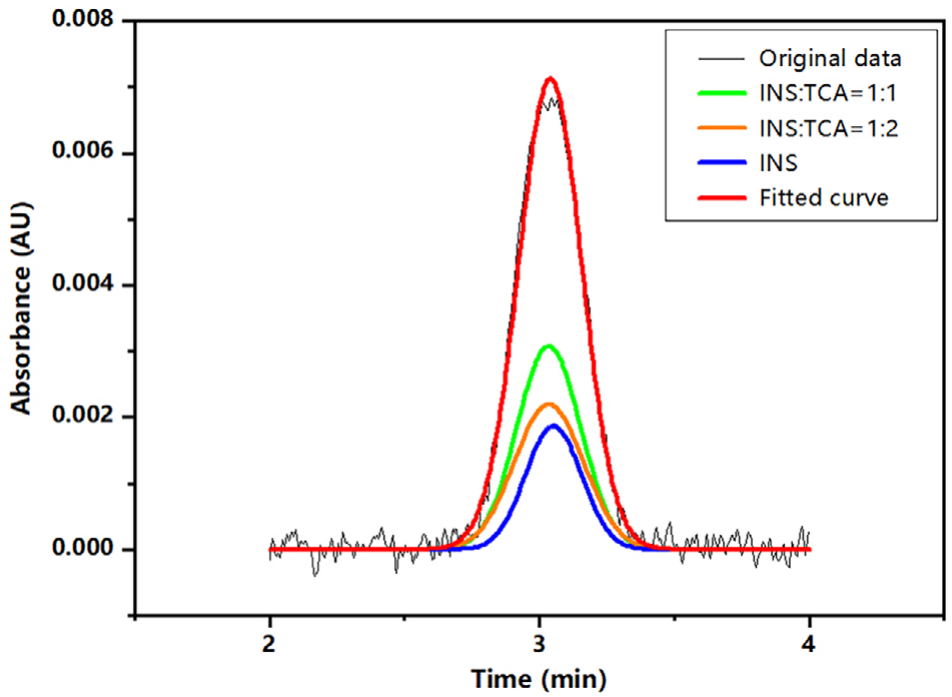
The Taylorgrams obtained from 300 µM insulin mixed with 58 mM TCA at pH 3.5, shown as the experimental data (black line) and Gaussian fitting (red line). The green, orange, and blue lines are the individual Gaussian peaks obtained by deconvolution, corresponding to the 1:1 and 1:2 INS‐TCA complexes, and the free insulin, respectively.

We tested the effects of TCA binding on the Taylorgram of INS. For INS (300 µM) in an acid pH solution, the Taylorgram displayed a Gaussian shape (Figure S1). Through Gaussian fitting, the diffusion coefficient of INS was determined to be 1.04 × 10^−10^ m^2^/s, with the corresponding hydrodynamic radius (2.35 ± 0.03) nm obtained. Next, TDA was performed for a preincubated sample of 300 µM INS mixed with 58 mM TCA. The binding of TCA to INS occurred in the sample plug. The advantage of the TDA method lies in its ability to produce a Taylorgram for mixture samples, which is a convolution of individual Gaussian peaks. The resulting Taylorgram showed a convolution of individual Gaussian peaks, indicating the presence of various INS species and their complexes (Figure 6). Each of these peaks corresponded to a specific diffusion coefficient. From the Taylorgrams, we can obtain the migration time and temporal variance of each individual peak, which can be modeled using a Gaussian function. The three distinct peaks obtained by deconvolution correspond to the coexistence of INS‐TCA complexes and free INS. The variation in the temporal variance, as reflected in the peak width, provided a means to effectively identify individual INS species. *R* ^2^ values obtained from fitting individual peaks with a Gaussian function are typically above 0.99, indicating that the measurement quality of the resulting symmetrical Taylorgrams is satisfactory. However, there were slight shifts in the migration times of individual peaks from the Taylorgrams, measured at 3.05, 3.03, and 3.03 min, respectively. These peaks correspond to the coexisting free INS, as well as the 1:1 and 1:2 INS‐TCA complexes in the mixture. The minor changes in migration times may suggest a small amount of INS adsorption onto the capillary wall [45].

The *D* values of these species are listed in Table 1. The hydrodynamic radius *R*_h_ can be determined based on the Stokes–Einstein relationship (Equation 2). Curve fitting of the Gaussian peaks revealed diffusion coefficients of 7.22 × 10^−11^ m^2^/s and 5.71 × 10^−11^ m^2^/s, with corresponding *R*_h_ values of (3.39 ± 0.03) nm and (4.28 ± 0.18) nm, which correspond to the sizes of the 1:1 and 1:2 INS‐TCA complexes, respectively. Notably, the radius of the free INS (2.37 ± 0.17 nm) in the mixed solution closely matches that in the INS solution (in the absence of TCA). The TDA results demonstrated that this technique can rapidly identify INS variations in mixtures without requiring pretreatment for separation. TCA binding was observed to increase INS size in both the 1:1 and 1:2 INS‐TCA complexes, indicating that the binding sites are probably located on the surface of the INS molecules.

**TABLE 1:** Diffusion coefficients and hydrodynamic radii of INS and its complexes obtained by TDA method.

	Diffusion coefficient, *D* (m^2^/s)	Hydrodynamic radius, *R*_h_ (nm)
Free INS	1.05 × 10^−10^	2.37 ± 0.17
1:1 INS‐TCA complex	7.22 × 10^−11^	3.39 ± 0.03
1:2 INS‐TCA complex	5.74 × 10^−11^	4.28 ± 0.18

### 3.5 Investigation of the Binding Characteristics of INS‐TCA Interactions by PACE‐FA

Although the ESI‐MS results revealed multiple binding stoichiometries of TCA toward INS and confirmed the stability of the major 1:1 INS‐TCA complex in the gas phase, the stoichiometry of the native‐state binding pair in solution, along with its binding constants, requires further investigation. If the binding interaction is measured in the liquid phase using a solution‐based method such as CE, the experimental results may vary from the ESI‐MS results to some extent. Owing to the lack of solvents in the gas phase, hydrogen bonding and electrostatic forces play major roles. However, in solution, hydrophobic interactions between the receptors and ligands cannot be ignored.

The PACE‐FA method was employed to analyze the solution‐state binding interactions. The signal of the TCA molecules, acting as the ligand, exhibited a plateau shape, with the height of the signal correlating to the concentration of the unbound ligand in the equilibrated system. To reduce the degree of adsorption of interacting species onto the capillary wall, a neutral‐coated capillary was used in the CE‐FA experiments. However, it is not feasible to allow all interacting species, particularly those with opposite charges (such as INS and TCA), to migrate using only the electrical potential applied to the capillary. Optimal detection was obtained at acidic pH of 3.5 (15 mM NaH_2_PO_4_‐H_3_PO_4_) above p*K*_a_ of TCA (1.4) where it was negatively charged. In this buffer, INS was positively charged due to its isoelectric point of 5.4 [46]. All analytes were detected using PACE‐FA. Additionally, applying a controlled hydrodynamic pressure (≤2 psi) during the CE‐FA process helped reduce the time of binding analysis while maintaining measurement accuracy. As the ligand concentration increased in the PACE‐FA binding assay, the number of ligands bound to each host molecule (*I*) also increased. This relationship can be described by Equation (6), which can be used to measure the binding interaction, even when the stoichiometry is uncertain [47, 48]:

(6) $$I=\frac{C_{b}}{H_{t}}=\frac{nKC_{f}}{1+KC_{f}}\;$$

where *C*_b_ and *H*_t_ denote the bound TCA and total INS concentrations, respectively. *I* indicates the average binding ratio and reflects whether all binding sites on INS are saturated by TCA. *C*_f_ denotes the unbound TCA concentration, which is determined using a standard calibration curve. Here, *C*_b_ was derived by subtracting *C*_f_ from the total TCA concentration. Nonlinear regression was employed to determine the binding parameters, including the binding constant (*K*) and binding stoichiometry (*n*), using Equation (6).

**FIGURE 7:**
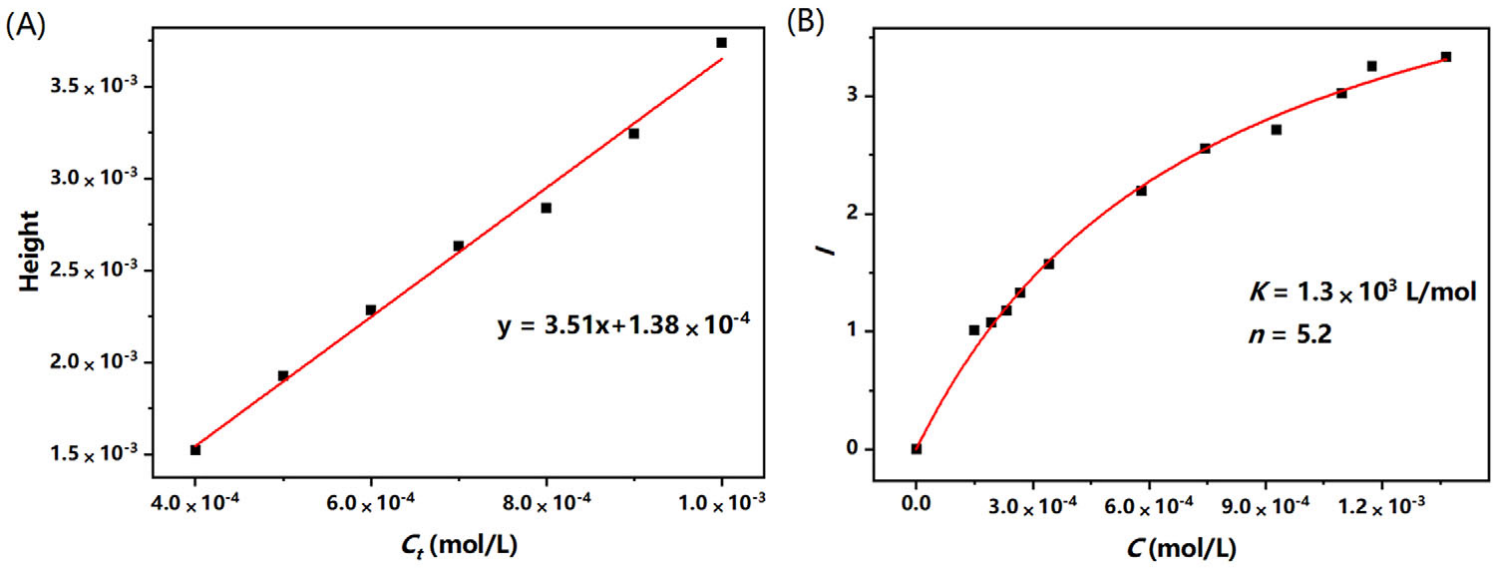
(A) The standard calibration curve of TCA; (B) the binding isotherm of INS with TCA via PACE‐FA.

Owing to the difference in mobility between free TCA and the INS‐TCA complex, the free TCA zone could be separated from the complex zone, forming a plateau peak. A typical PACE‐FA electropherogram of the INS‐TCA sample is shown in Figure S2. To successfully conduct the PACE‐FA binding assay, it is essential to optimize the voltage, external pressure, and concentration of the NaH_2_PO_4_‐H_3_PO_4_ buffer. The optimal plateau shape and stability were obtained using a +15 kV voltage assisted by +0.7 psi (+4.83 kPa) pressure, with a 15 mM NaH_2_PO_4_‐H_3_PO_4_ buffer selected for the binding studies. The experimental conditions for the optimized PACE‐FA method are shown in Figure S2. Under these optimized conditions, samples containing TCA (4.0 × 10^−4^–1.0 × 10^−3^ M) standards were used to create the calibration curve (Figure 7A). Mixtures containing 100 µM INS and increasing concentration of TCA were used for determining the binding constant *K*. From Figure 7B, the binding constant of the interaction between INS and TCA was computed to be (1.3 ± 0.1) × 10^3^ L/mol (*R* ^2^ = 0.994) based on nonlinear regression analysis. The number of binding sites for TCA on INS (5.2 ± 0.1) closely matched the initial ESI‐MS results.

### 3.6 Interaction Between INS and TCA: Results From Computer Simulations

**FIGURE 8:**
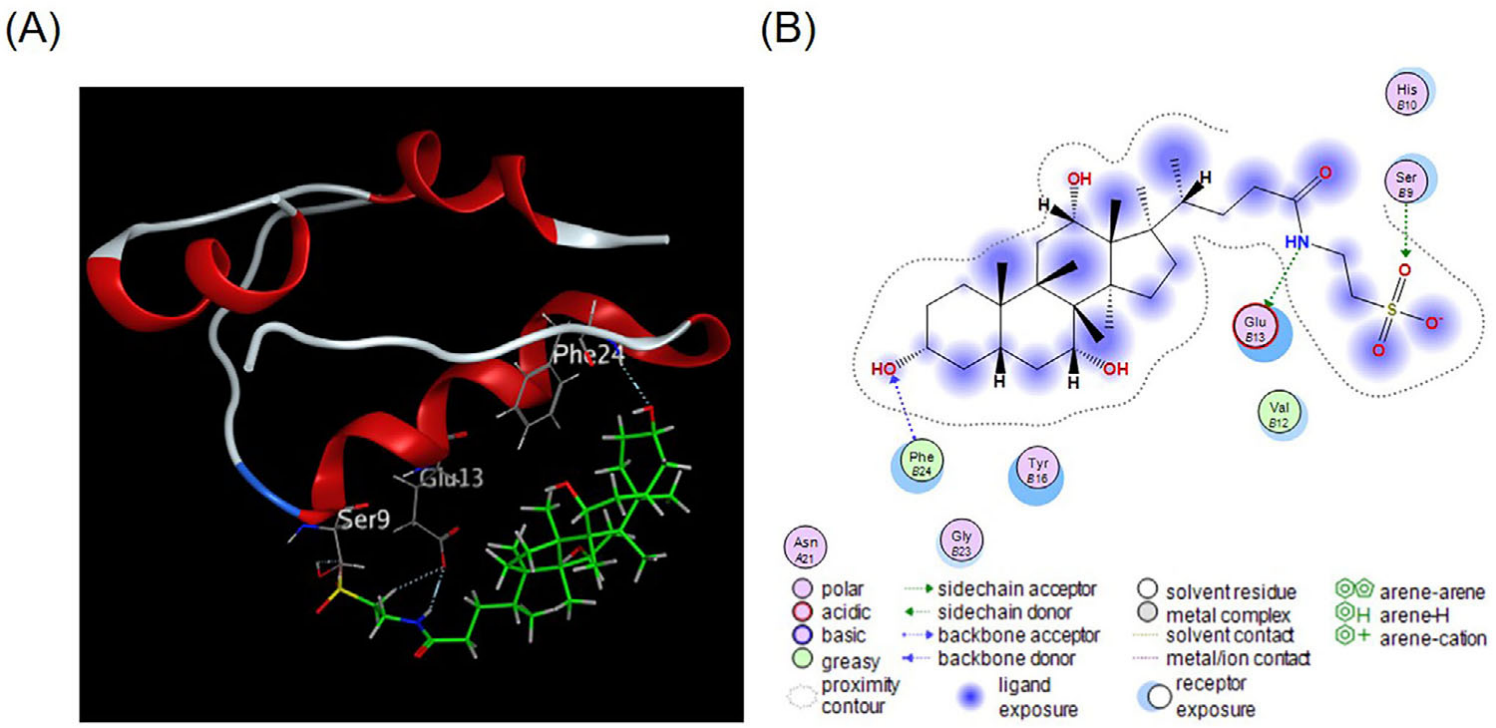
(A) Docked location of TCA (green) with key residues in insulin (red and gray); (B) the hydrogen bonds (arrows with dashed lines) of TCA with amino acid residues of the insulin.

Molecular docking was used to complement the experimental measurements to probe the binding sites and modes of TCA on INS. Human INS contains two polypeptide chains A and B linked by two interchain disulfide bonds (A7‐B7 and A20‐B19). The A chain contains 21 amino acids, with an intrachain disulfide bond (A6‐A11), while the B chain contains 30 amino acids [49]. The dominant conformation of the INS‐TCA complex is shown in Figure 8A. Docking results revealed that TCA mainly utilizes hydrogen bonds to bind to the B chain of the INS molecule. Specifically, three hydrogen bonds were formed between INS's B‐chain α‐helix (B9‐B19) [50] and TCA (Ser‐B9, Glu‐B13, and Phe‐B24 residues), with docking energies of −1.2, −4.4, and −1.3 kcal/mol, respectively (Figure 8B). These hydrogen bonds likely play a key role in the formation of the INS‐TCA complex. The molecular docking results also indicated that the TCA binds to an external binding site on INS near the Ser‐B9, Glu‐B13, and Phe‐B24 residues, which aligns with the ESI‐MS and TDA results.

## 4 Concluding Remarks

In this study, the binding capacity and stoichiometry of TCA with INS were rapidly evaluated using ESI‐MS and PACE‐FA. TCA, as an absorbent for oral INS therapy, likely facilitates the formation of INS‐TCA non‐covalent complexes [30, 51]. ESI‐MS and PACE‐FA results indicated that the binding constant of TCA to INS was 1.3 × 10^3^ L/mol, with TCA binding to INS at five sites. The binding did not change the secondary structure of INS; however, the formation of the INS‐TCA complex improved the structural stability of INS. The increased radii of the complexed molecules further supported the TDA results. Computational simulations revealed that TCA primarily utilizes three hydrogen bonds to bind to the external binding site on the B chain of INS. Therefore, the enhanced absorption of INS through binding to TCA, a previously unrecognized factor, is expected to contribute to the improved bioavailability of oral INS therapy. We believe that the method described in this work can be applied to evaluate other molecular interactions for drug delivery system and pharmaco‐activity study.

## Conflicts of Interest

The authors declare no conflicts of interest.

## Supporting information



Supporting Information

## Data Availability

All data included in this study are available upon request by contact with the corresponding authors.
