# Supplementary material for: Characterization of Taurocholic Acid Binding With Insulin for Potential Oral Formulation Using Different Methods
Source: Electrophoresis. 2025 Apr 16;46(7-8):468–77. doi: 10.1002/elps.8139 (PMC12039169; doi:10.1002/elps.8139)
Supplement: Supplementary file 1 — Supporting Information [file ELPS-46--s001.docx]

**Supporting Information**

**Characterization of Taurocholic Acid Binding with Insulin for Potential Oral Formulation Using Different Methods**

Chang Sun,^1^ Shuanghao Wang,^1,2^ Huihui Li,^1,3,*^ David Da Yong Chen^4,*^

*^1^State Key Laboratory of Analytical Chemistry for Life Science*，*National and Local Joint Engineering Research Center of Biomedical Functional Materials, Jiangsu Collaborative Innovation Center of Biomedical Functional Materials, Changzhou Institute of Innovation and Development, School of Chemistry and Materials Science, Nanjing Normal University, Nanjing 210023, P. R. China*

^2^ *Jiangyan High School of Jiangsu Province, Taizhou 225599, P. R. China*

^3^ *Jiangsu Key Laboratory for Biosensors, Institute of Advanced Materials (IAM), Nanjing University of Posts & Telecommunications, 9 Wenyuan Road, Nanjing 210023, P. R. China*

^4^ *Department of Chemistry, University of British Columbia, Vancouver, BC, Canada V6T 1Z1*

*Correspondence should be addressed to:

Prof. Huihui Li: [huihuili@njnu.edu.cn](mailto:huihuili@njnu.edu.cn)

Prof. David D. Y. Chen: [chen@chem.ubc.ca](mailto:chen@chem.ubc.ca)

Abbreviations: CD, Circular dichroism; INS, insulin; MOE, Molecular Operating Environment; PACE-FA, pressure-assisted capillary electrophoresis frontal analysis; PDB, Protein Data Bank; TCA, taurocholic acid; TDA, Taylor dispersion analysis

Keywords: capillary electrophoresis, insulin, interaction, mass spectrometry, taurocholic acid

Table S1. The obtained data from TDA analyses of Insulin at different mobilization pressures

| *P* (psi) | *u* (m/s) | *t*_d_ (s) | *D* (m^2^/s) | SD (m^2^/s) | *P*_e_ | *R*^2^ |
| --- | --- | --- | --- | --- | --- | --- |
| 1 | 1.00×10^-3^ | 506 | 9.21×10^-11^ | 5.48×10^-13^ | 273 | 0.998 |
| 2 | 2.01×10^-3^ | 254 | 9.74×10^-11^ | 6.16×10^-13^ | 516 | 0.998 |
| 3 | 3.01×10^-3^ | 171 | 1.01×10^-10^ | 8.49×10^-13^ | 747 | 0.998 |
| 4 | 4.02×10^-3^ | 130 | 1.03×10^-10^ | 7.67×10^-13^ | 974 | 0.998 |
| 5 | 5.02×10^-3^ | 105 | 1.06×10^-10^ | 9.93×10^-13^ | 1184 | 0.998 |

*P*: mobilization pressures; SD: standard deviations in the diffusion coefficient within a set of 4 replicate runs; *R*^2^: calculated by fitting the elution peak by a Gaussian function.

Table S2. The obtained data from TDA analyses of insulin using capillaries with different lengths

| *L* (cm) | *t*_d_ (s) | *D* (m^2^/s) | SD (m^2^/s) | *P*_e_ | *R*^2^ |
| --- | --- | --- | --- | --- | --- |
| 50 | 163 | 9.81×10^-11^ | 1.73×10^-13^ | 614 | 0.999 |
| 60 | 254 | 9.74×10^-11^ | 6.16×10^-13^ | 516 | 0.998 |
| 70 | 357 | 9.72×10^-11^ | 9.38×10^-13^ | 443 | 0.998 |

Figure S1. The Taylorgram obtained from 300 µM insulin at pH 3.5 depicting the experimental data (black line) and the Gaussian fit (red line).

(A) (B)


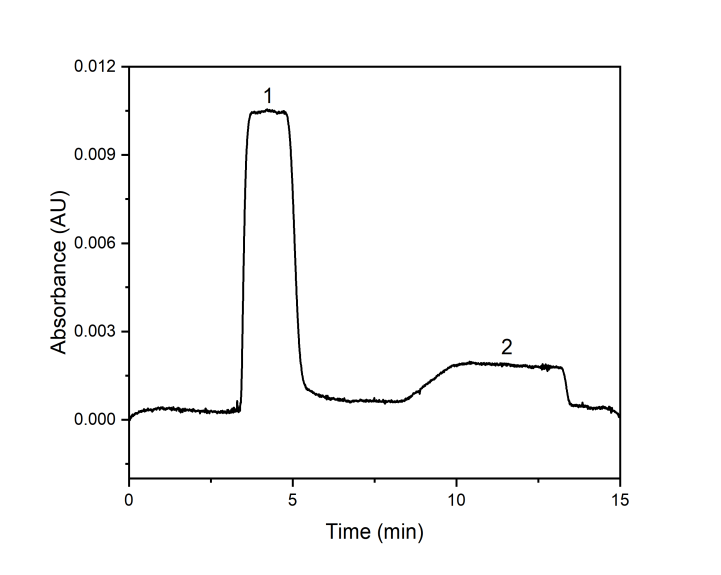

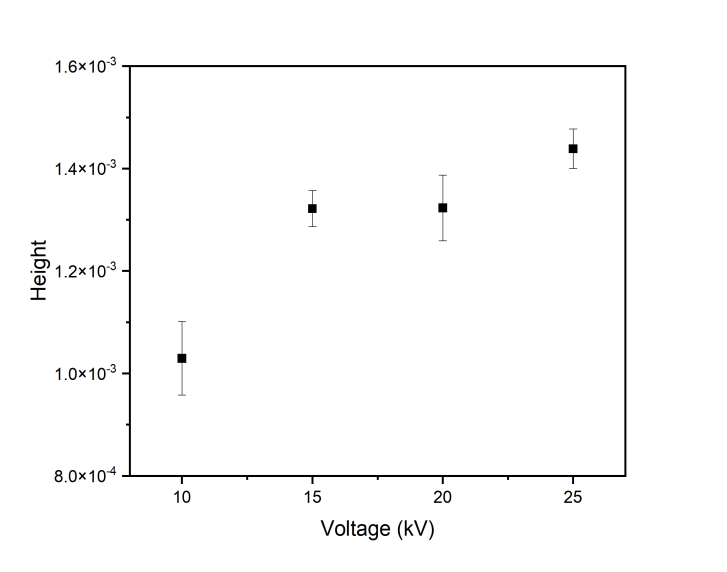


(C) (D)


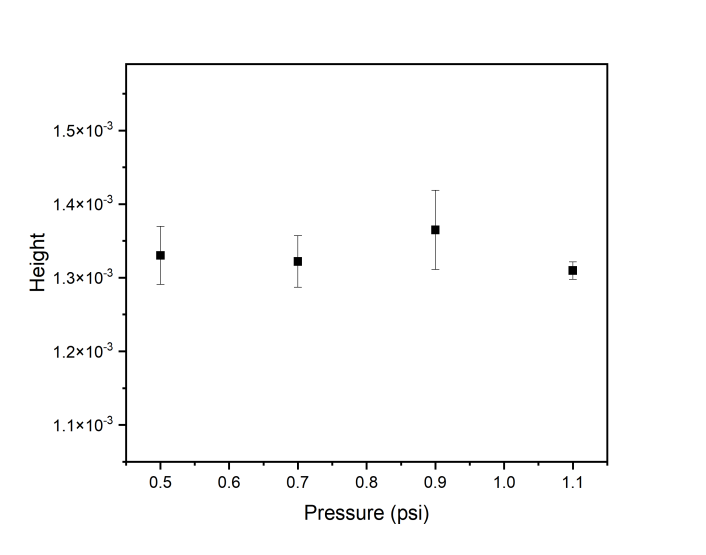

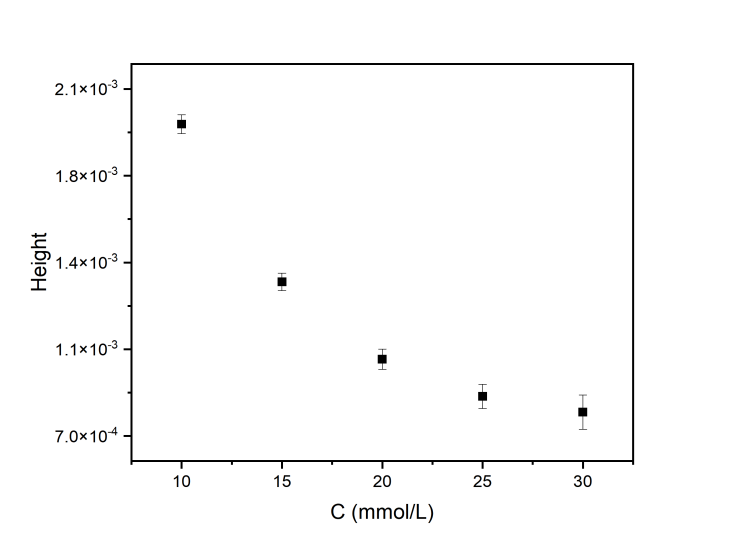


Figure S2. (A) The PACE-FA electropherogram of INS-TCA mixture in the molar ratio of 1:10 in 15 mM NaH_2_PO_4_-H_3_PO_4_ (pH 3.5). 1 and 2 represent the peak of insulin and TCA, respectively. The optimization for PACE-FA within a set of 3 replicate runs. (B) the voltage, (C) the external pressure and (D) the concentration of NaH_2_PO_4_-H_3_PO_4_ buffer.
